# Selective killing of gastric cancer cells by a small molecule via targeting TrxR1 and ROS-mediated ER stress activation

**DOI:** 10.18632/oncotarget.7565

**Published:** 2016-02-22

**Authors:** Weiqian Chen, Peng Zou, Zhongwei Zhao, Qiaoyou Weng, Xi Chen, Shilong Ying, Qingqing Ye, Zhe Wang, Jiansong Ji, Guang Liang

**Affiliations:** ^1^ Chemical Biology Research Center, School of Pharmaceutical Sciences, Wenzhou Medical University, Wenzhou, Zhejiang, 325035, China; ^2^ Department of Interventional Radiology, The Fifth Affiliated Hospital of Wenzhou Medical University, Lishui, Zhejiang, 323000, China; ^3^ School of Environmental and Biological Engineering, Nanjing University of Science and Technology, Nanjing, Jiangsu, 210094, China

**Keywords:** thioredoxin reductase 1, reactive oxygen species (ROS), gastric cancer, ER stress, mitochondrial dysfunction

## Abstract

The thioredoxin reductase (TrxR) 1 is often overexpressed in numerous cancer cells. Targeting TrxR1 leads to a reduction in tumor progression and metastasis, making the enzyme an attractive target for cancer treatment. Our previous research revealed that the curcumin derivative B19 could induce cancer cell apoptosis via activation of endoplasmic reticulum (ER) stress. However, the upstream mechanism and molecular target of B19 is still unclear. In this study, we demonstrate that B19 directly inhibits TrxR1 enzyme activity to elevate oxidative stress and then induce ROS-mediated ER Stress and mitochondrial dysfunction, subsequently resulting in cell cycle arrest and apoptosis in human gastric cancer cells. A computer-assistant docking showed that B19 may bind TrxR1 protein via formation of a covalent bond with the residue Cys-498. Blockage of ROS production totally reversed B19-induced anti-cancer actions. In addition, the results of xenograft experiments in mice were highly consistent with in vitro studies. Taken together, targeting TrxR1 with B19 provides deep insight into the understanding of how B19 exerts its anticancer effects. More importantly, this work indicates that targeting TrxR1 and manipulating ROS levels are effective therapeutic strategy for the treatment of gastric cancer.

## INTRODUCTION

Redox homeostasis, the balance of which is regulated by two major cellular antioxidant systems (the glutathione system and the thioredoxin system), plays a crucial role in cellular viability and function [1–3]. Reactive oxygen species (ROS) are normal byproducts of numerous cellular processes, such as mitochondrial metabolism and protein folding [4]. As compared to normal cells, cancer cells usually possess higher levels of ROS and higher antioxidant activities in an uncontrolled status [5]. As a result, cancer cells are unable to cope with additional oxidative stress and become vulnerable to excessive ROS [6]. Therefore, overproducted ROS disrupts the intracellular redox balance and exerts an oxidative stress on cancer cells that can ultimately cause cell senescence or death [7].

The thioredoxin system, which consists of thioredoxin (Trx), thioredoxin reductase (TrxR) and nicotinamide adenine dinucleotide phosphate (NADPH), plays an important role in redox signal transduction for cell growth and apoptosis [8–11]. Besides the redox regulation of intracellular signaling, the system also exerts its direct antioxidant defense against oxidative stress, including scavenging reactive oxygen species (ROS) [12], reducing peroxides [13] and endogenous antioxidant recycling [14]. Two major isoforms of TrxR/Trx are present in different intracellular organelles: TrxR1/Trx1 are predominantly in the cytosol and nucleus, while TrxR2/Trx2 are mainly localized within mitochondrions [15]. Given that TrxR1 is often overexpressed in many cancer cells and targeting its ablation leads to a reduction in tumor progression and metastasis, this selenoenzyme is highlighted as a potential target for cancer therapy via regulating redox balance in cancer cells [16, 17]. Several effective natural and synthetic TrxR1 inhibitors are now available, possessing antitumor potential ranging from induction of Reactive oxygen species (ROS) to cell cycle arrest and apoptosis [17–19].

**Figure 1:**
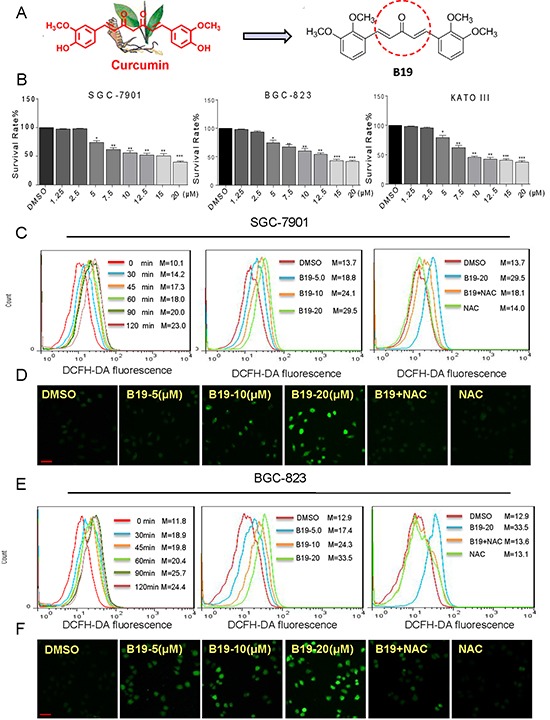
B19 inhibits cells growth and induces accumulation of Intracellular ROS in human gastric cancer cells **A.** Chemical structure of curcumin and B19. **B.** Effects of B19 in human gastric cancer cell viability. Cells were treated with concentration gradient of B19 for 24h and processed for MTT assay. **C, E.** Intracellular ROS generation time- and dose-dependently induced by B19 was measured in SGC-7901 cells and BGC-823 cells by staining with DCFH-DA (10 μM) and flow cytometry analysis. SGC-7901 cells and BGC-823 cells were treated with B19 (20 μM) for the indicated times. SGC-7901 cells and BGC-823 cells were pre-incubated with or without 5 mM NAC for 2 h before exposure to B19 at the indicated concentrations for 30 min. Then, Intracellular ROS generation was measured by flow cytometry. **D, F.** Intracellular ROS generation induced by increasing doses of B19 for 30 min was measured in SGC-7901 cells and BGC-823 cells by fluorescence microscope. SGC-7901 cells and BGC-823 cells were pre-incubated with 5 mM NAC for 2 h before exposure to B19 (20 μM) for 30 min. Intracellular ROS generation was measured by fluorescence microscope. A scale bar, 20μm. All images shown here are representative of three independent experiments with similar results. Error bars represent S.E.M. of triplicates (*P< 0.05, **P < 0.01, ***P < 0.001).

In our previous study, a monocarbonyl analogue of curcumin, (1E, 4E)-1, 5-bis (2-methoxyphenyl) penta-1, 4-dien-3-one (B19, Figure 1A), has been designed and found as an anti-tumor agent [20]. We have demonstrated that B19 could induce lung cancer cell apoptosis via activation of ER stress [20]. Although this molecule is now being full evaluated in anti-cancer pre-clinical studies, the upstream target and mechanism by which B19 induced ER stress-dependent apoptosis are still unknown, which even limits the drug development of B19 and its derivatives.

Gastric cancer (GC) is the fourth most commonly diagnosed cancer and the second leading cause of cancer-related death in the world [21]. Only 20% of the patients are suitable for curative resection because the majority of patients are initially diagnosed with the advanced-stage gastric cancer [22]. The need for the new therapeutic strategies and new adjuvant agents is especially urgent. In this paper, we provided evidence that B19 targets TrxR1 to induce oxidative stress in human gastric cancer cells. Our observations also discovered that B19 induces apoptotic cell death in gastric cancer cells, via activating ROS-dependent ER stress and mitochondrial pathways, blockage of ROS production by specific inhibitor totally abolished the anti-cancer effects of B19. Knockdown of the enzyme in cells enhances the cyto-toxicity of B19. Combined with the results of in vivo experiments, our findings provided a molecular mechanism by which B19 induces ROS-mediated apoptosis in cancer cells, and shed light in understanding how B19 works in vivo.

## RESULTS

### B19 effectively suppresses the proliferation and increases ROS levelsin human gastric cancer cells

To evaluate the effect of B19 on human gastric cancer cell lines (SGC-7901, BGC-823 and KATO III), the cells were treated with increasing concentrations of B19 for 24 h and then examined cell viability using the MTT assay. As show in Figure 1B, treated with B19 for 24 h significantly induced cell death in a dose-dependent manner in the three gastric cancer cells respectively (IC_50_ = 13.9, 14.2, and 13.1μM). We next examined whether the killing effect of B19 on gastric cancer cells was associated with increasing ROS levels. ROS levels in human gastric cancer cells (SGC-7901 and BGC-823) were assessed by flow cytometry using the redox-sensitive fluorescent probe 2′-,7′-dichlorofluorescein diacetate (DCFH-DA). As shown in Figure 1C and 1E, treatment with B19 in SGC-7901 cells and BGC-823 cells caused a time-dependent and dose-dependent increase in ROS levels, which indicated that B19 could interfere with the levels of intracellular ROS. However, pre-treated with the NAC, a specific ROS inhibitor, for 2h significantly inhibited the B19-induced increase in ROS levels. Meanwhile, the fluorescence intensity detected by a fluorescence microscope also showed that B19 could increase the levels of intracellular ROS in dose-dependent manner and this increase was fully reversed by pretreatment of the cells with NAC (Figure 1D and 1F). These results revealed that B19 could induce ROS accumulation and cell death in gastric cancer cells.

### B19 induces ROS-dependent apoptosis in human gastric cancer cells

**Figure 2:**
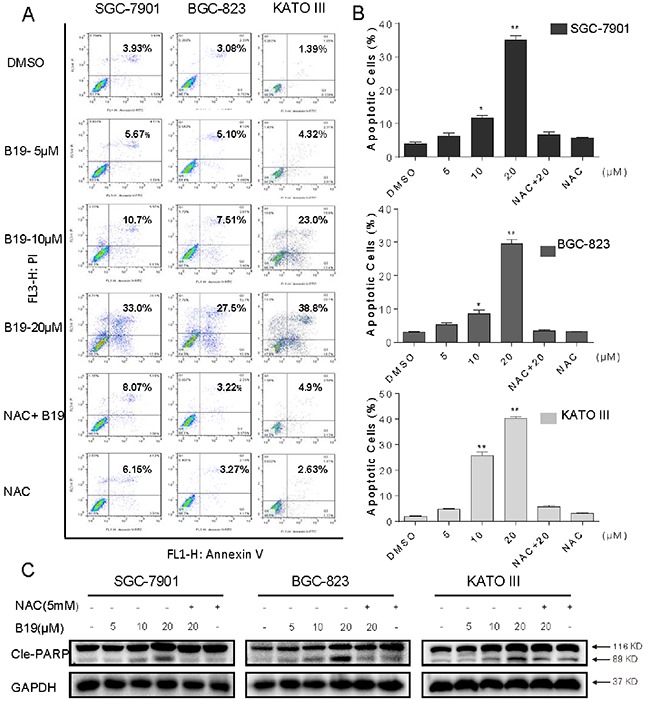
B19 induces ROS-dependent apoptosis in human gastric cancer cells **A.** SGC-7901, BGC-823 or KATO III cells were pre-incubated with or without 5 mM NAC for 2 h before exposure to B19 at the indicated concentrations for 24 h. Percentage of cell apoptosis was determined by Annexin-V/PI staining and flow cytometry. Similar results were obtained in three independent experiments. **B.** The percentage of apoptotic cells in the treatment groups was calculated. **C.** Three gastric cancer cells were pre-incubated with or without 5 mM NAC for 2 h before exposure to B19 at indicated concentration for 24h, the Cle-PARP expression were determined by western blot. GAPDH was used as internal control. All data here are expressed as means±S.D. of triplicates. All images shown here are representative of three independent experiments with similar results. Error bars represent S.E.M. of triplicates (*P< 0.05, **p < 0.01).

We further examined the pro-apoptotic effect of B19 inhuman gastric cancer cell lines, using Annexin V/propidium iodide (PI) staining assay. To identify the role of ROS in mediating B19's anti-cancer effects, NAC was used. As shown in Figure 2A and 2B, all of three gastric cancer cell lines have shown a concentration-dependent apoptotic cell death after a 24 h treatment with B19. Interestingly, NAC almost completely abolished cancer cell apoptosis induced by B19. PARP cleavage seems to be involved in the repair of DNA damage in response to apoptotic signal stimulation and serves as a marker of apoptosis. We therefore examined the effects of B19 on PARP cleavage in the presence or absence of NAC (5 mM), using western blot assay. As shown in Figure 2C, B19 dose-dependently increased the expression of Cle-PARP in all three gastric cancer cell lines. While, pre-treatment with NAC at 5 mM fully reversed the B19-induced increase in PARP cleavage. These results verified that ROS induction mediates B19-activated apoptotic path ways and is critical upstream regulator in B19's anti-cancer activity.

### B19 induces ROS-dependent G2/M cell cycle arrest in human gastric cancer cells

**Figure 3:**
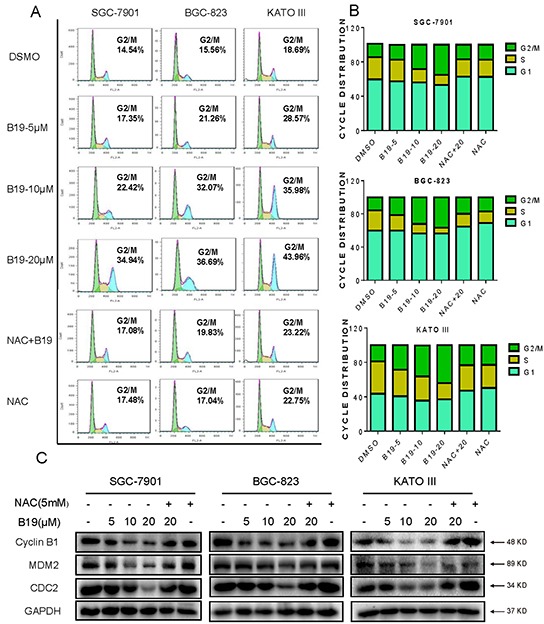
B19 induces ROS-dependent cell cycle arrest via in human gastric cancer cells **A.** SGC-7901, BGC-823 or KATO III cells were pre-incubated with or without 5 mM NAC for 2 h before exposure to B19 at the indicated concentrations for 15 h. The number of cells in G2/M phase was determined via flow cytometry. **B.** Representative histograms from flow cytometry analysis in the three human gastric cancer cells treated with B19 in the presence or absence of NAC. Assays were performed in triplicate. **C.** Expression of G2/M cell cycle relative proteins MDM-2, Cyclin B1 and Cdc2 were determined by western blot after treatment with B19 (5, 10 or 20 μM) or B19 (20 μM)+NAC (5 mM) pretreated or NAC (5 mM) for 15 h in three gastric cancer cells. GAPDH was used as internal control.

To determine whether the cell cycle arrest is involved in B19-caused growth inhibition in gastric cancer cells, cells were pre-incubated with NAC (5 mM) for 2 h before exposure to increasing concentrations of B19 for 24h, followed by the cell cycle determination by flow cytometry. The results in Figure 3A and 3B showed that B19 dose-dependently induces G2/M cell cycle arrest, while blocking of ROS generation by NAC totally reversed the B19-induced G2/M cell cycle arrest in human gastric cancer cells. Western blotting analysis also indicated that B19 dose-dependently inhibited the expression of cell cycle-related proteins such as MDM-2, Cyclin B1 and Cdc2 in human gastric cancer cells (Figure 3C). Consistent with the flow cytometry outcomes, NAC significantly prevented the down-regulation of cell cycle-related proteins induced by B19. These results suggested that the inhibition of cell proliferation by B19 may be partly associated with the induction of G2/M phase arrest and that ROS induction also mediates B19-induced G2/M phase cell cycle arrest.

### B19 targets TrxR1 and inhibits its catalytic activity **in vitro**

**Figure 4:**
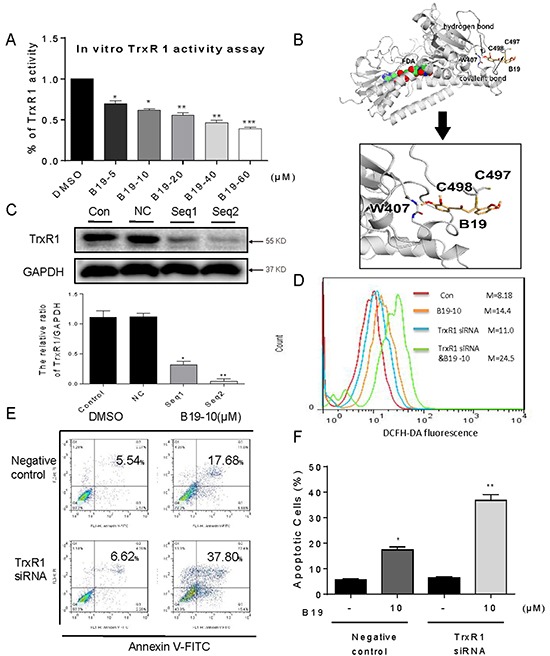
B19 targets and inactivates TrxR1 in human gastric cancer cells **A.** TrxR1 enzyme activity was measured with/without B19 treatment in vitro. **B.** Molecular docking of B19 with TrxR1 protein was carried out with the program Tripos molecular modeling packages Sybyl-x.v1.1.083. **C.** The TrxR1 expression was determined by Western blotting after knockdown with two different siRNAs for 48 h. Western blot results were calculated and compared with control. **D-E.** Knockdown of TrxR1in SGC-7901 cells significantly alter the ROS levels (D) and apoptotic cells (E) induced by B19. **F.** The percentage of apoptotic cells in each group was calculated. All images shown here are representative of three independent experiments with similar results. Error bars represent S.E.M. of triplicates (*P< 0.05, **P < 0.01, ***P < 0.001).

All of these observations above prompted us to identify the potential target of B19. It has been reported that TrxR1 interacts with a number of naturally occurring or synthetic compounds, such as curcumin [23], indolequinones [11], juglone [24], pleurotin [25], pyrroloquinolinequinone [26], anddoxorubicin [27]. Based on the structure of B19 and the ability to increasing ROS, we speculated that B19 might target TrxR1. We investigated the inhibitory activity of B19 on TrxR1 using the endpoint insulin reduction assay in vitro. As shown in Figure 4A, TrxR1 activity in cell lysates significantly decreased on treatment with B19 in a dose-dependent manner. We tried to see the possible binding sites of B19 in TrxR1 protein using the molecular docking method. Figure 4B showed that B19 inserts into the TrxR1. The Michael acceptor in intermediate chain of B19 can form a covalent bond with the residue Cys-498 of the C-terminal active site redox center of TrxR1, and the carbonyl oxygen atom forms hydrogen bonds with the main-chain of residue W407. It was reported that cysteine and selenocysteine residues in the redox motifs of TrxR1 are both critical for enzyme inactivation [23, 28–30]. Although our simulation showed Cys-498, rather than Sec-498, plays a role in the interaction between B19 and TrxR1, the exact binding mode in cancer cells needs to be demonstrated by bioengineering technique in the future. These data indicate that B19 could target TrxR1 protein and inhibit TrxR1 enzyme activity.

To further elucidate the role of TrxR1 in B19-induced ROS accumulation and apoptosis, we constructed two separate TrxR1 siRNA for silencing TrxR1 expression in SGC-7901 cells. The reduction of TrxR1 expression by Seq1 and Seq2 siRNAs in SGC-7901 cells was confirmed by Western blot assay (Figure 4C). Seq2 exhibited a better gene silencing efficacy than Seq1. As shown in Figure 4D, knockdown of TrxR1 by Seq2 increased B19-induced ROS accumulation. Similarly, TrxR1 silencing also significantly increased B19-induced apoptosis in SGC-7901 cells (Figure 4E and 4F). Taken together, these results solidly supported our hypothesis that B19 targets TrxR1 to elevate oxidative stress and subsequently induce apoptosis in human gastric cancer cells.

### B19 activates ER stress signaling via promoting the accumulation of ROS in human gastric cancer cells

**Figure 5:**
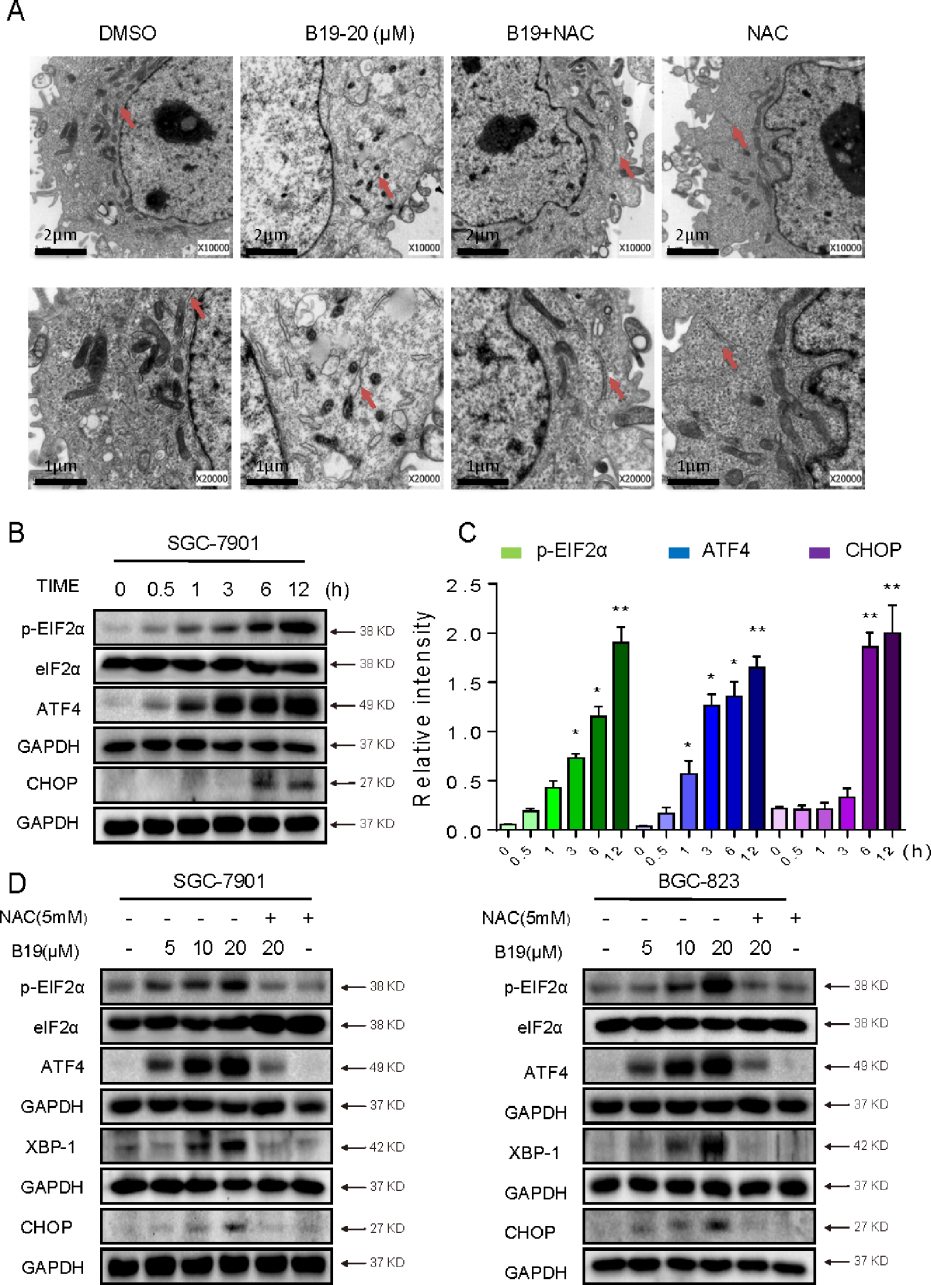
B19 is found to activate the ER stress via promoting the accumulation of ROS **A.** Effect of B19 on the morphology of endoplasmic reticulum in SGC-7901 cells. SGC-7901 cells were treated as described in Experimental Section. The morphology of endoplasmic reticulum in SGC-7901 cells was examined with an electron microscope (×10000 or ×20000). Results from a representative cell sample out of three studied in each group are shown. The ER morphologies are normal in the control group, the B19 (20 μM)+ NAC (5 mM) group and NAC (5 mM) group (arrows indicate normal ER). Exposure to 20 μM B19 for 12 h induced mitochondrial dysfunction in B19 (20 μM) group (arrows indicate swollen ER). **B.** SGC-7901 cells were treated with B19 (20 μM) for the indicated times, the protein levels of ATF4, p-EIF2α and CHOP were determined by western blot. GAPDH and eIF2α were used as internal control. **C.** Western blot results from (B) were calculated and compared with control. **D.** SGC-7901 cells and BGC-823 cells were pre-incubated with or without 5 mM NAC for 2 h before exposure to B19 at the indicated concentrations, 3 hours later the ATF4, p-EIF2α, and XBP-1 expression were detected by western blot. The protein level of CHOP was examined by western blot after treatment with B19, B19+NAC pretreated, or NAC alone for 12 h. GAPDH and eIF2α were used as internal control.

Our previous study demonstrated that B19 induces cancer cell apoptosis via activation of endoplasmic reticulum stress signaling pathway [20, 31]. Oxidative stress has been reported to activate ER stress-related apoptosis [32]. Therefore, we speculated that B19 activates ER stress via increasing ROS level. The effect of B19 on the morphology of ER in SGC-7901 cells was observed through electronic microscopy. As shown in Figure 5A (×10000 and ×20000 amplification, respectively), DMSO-treated SGC-7901 cells showed the familiar appearance of smooth ER (arrow). While 6 hour treatment with B19 (10 μM) made the ER in SGC-7901 cells swelling (arrow), suggesting the accumulation of misfolded protein in ER. Pretreatment of the cells with NAC (5 mM) fully recovered the ER with familiar morphology, and treatment of cells with NAC (5 mM) alone had no impact on the morphology of ER (Figure 5A). The results were further confirmed in the level of proteins. The time-course results indicated that B19 (20 μM) could induce ER stress-related proteins (p-EIF2α, ATF-4 and CHOP) in time-dependent manner (Figure 5B and 5C). B19 also showed dose-dependent induction of ER stress-related proteins expression, whileB19-induced changes in ER stress-related proteins (p-EIF2α, ATF-4, XPB1 and CHOP) were all reversed by NAC pretreatment (Figure 5D). These results suggested that ROS generation also mediates B19-activated ER-stress pathway.

### B19 induces ROS-dependent mitochondrial dysfunction through regulation of Bcl-2 family proteins

**Figure 6:**
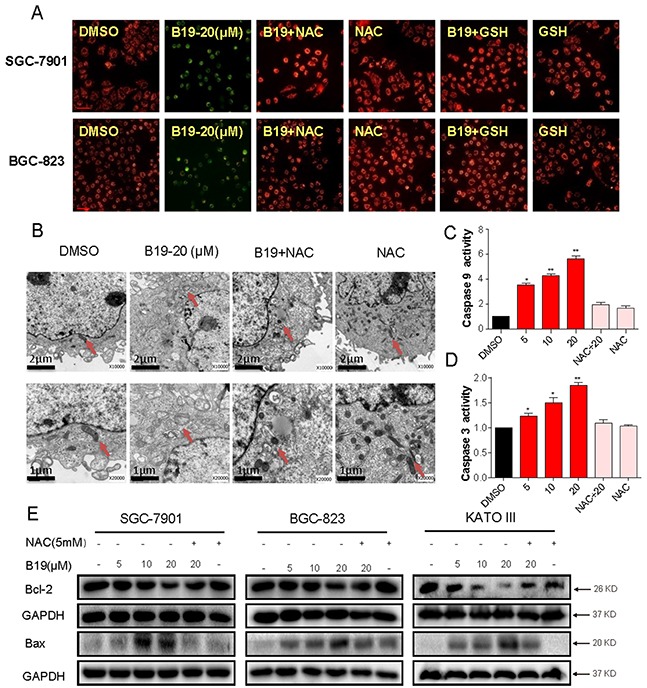
B19 reduces mitochondrial membrane potential (Δψm) and induces mitochondrial dysfunction via promoting the accumulation of ROS **A.** Mitochondrial membrane potential (Δψm) was detected by JC-1 kit. SGC-7901 cells and BGC-823 cells were pre-incubated with or without 5 mM NAC or 5 mM GSH for 2 h before exposure to B19 (20 μM) for 12h. After being stained by JC-1, mitochondrial membrane potential (Δψm) was detected using a fluorescence microscope. A scale bar, 20 μm. **B.** Effect of B19 on the morphology of mitochondria in SGC-7901 cells. SGC-7901 cells were treated as described in Experimental Section. The morphology of mitochondria in SGC-7901 cells was examined with an electron microscope (×10000 or ×20000). Results from a representative cell sample out of three studied in each group are shown. The mitochondria morphologies are normal in the control group, the B19 (20 μM)+ NAC (5 mM) group and NAC (5 mM) group (arrows indicate normal mitochondria). Exposure to 20 μM B19 for 12 h induced mitochondrial dysfunction in B19 (20 μM) group (arrows indicate swollen mitochondria). **C, D.** SGC-7901 cells were treated with B19 (5, 10 or 20 μM) in the presence or absence NAC(5mM) for 24h, the activity of caspase-9 and caspase-3 was determined by caspase-3 activity kit and caspase-9 activity kit. Data were analyzed and represented as the percentage of control. Data presented are representative of three independent experiments. **E.** Three gastric cancer cells were pre-incubated with or without 5 mM NAC for 2 h before exposure to B19 at indicated concentration for 24h, the Bcl-2 and Bax expression were determined by western blot. GAPDH was used as internal control. Error bars represent S.E.M. of triplicates(*P< 0.05, **P < 0.01).

It is well known that mitochondria are central to the regulation of apoptosis. Loss of mitochondrial membrane potential (Δ*ψ*_m_) is catastrophic for cells and leads to the release of cytochrome C into the cytosol. The next step is to investigate the effect of B19 on mitochondrial membrane potential by use of the potential-sensitive ratiometric fluorescence dye JC-1. As shown in Figure 6A, B19 (20 μM) caused a pronounced decrease of mitochondrial membrane potential (Δ*ψ*_m_), indicating a reduction of highly energized mitochondria. In contrast, the pretreatment with ROS inhibitors (NAC or GSH) for 2 h could attenuate the B19-induced decrease in mitochondrial membrane potential (Δ*ψ*_m_). To confirm the B19-induced mitochondrial swelling, we observed the mitochondrial features of SGC-7901 cells exposed to B19 (20 μM) for 12 h in the presence or absence of NAC, using electron microscopy. As shown in Figure 6B, the mitochondria in B19-treated cells appeared to be abnormally enlarged or swollen, revealing shredded cristae and the disruption of outer membrane integrity. Furthermore, pretreatment of the cells with NAC (5 mM) reversed the swelling of mitochondria induced by B19 (Figure 6B). Meanwhile, NAC alone had no impact on the morphology of mitochondria (Figure 6B).

**Figure 7:**
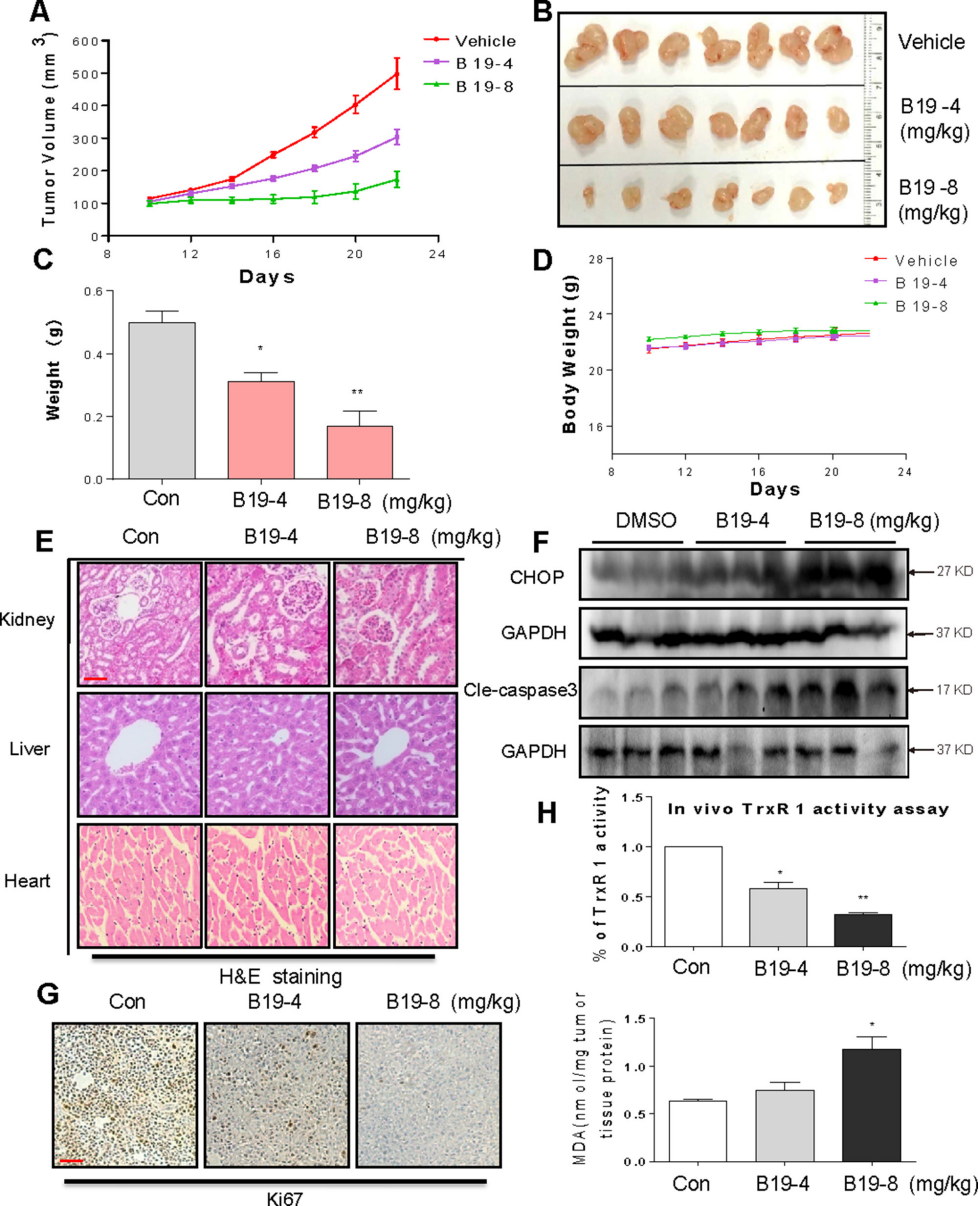
B19 inhibits gastric cancer tumor xenograft growth and induces oxidative injury in vivo B19 treatment dose-dependently inhibits tumor volume **A–B**. and tumor weight **C.** of SGC-7901 human gastric cancer xenografts in nude mice, but do not affect body weight **D.** of mice. **E.** Kidneys, livers and hearts tissues from 3 groups were sectioned at 5 μm and the slides were stained with hematoxylin and eosin (H&E), (n=5 in each group). All images were obtained by microscope with 20× magnification. B19 administration does not cause histological abnormalities in kidneys, livers and hearts. A scale bar, 50μm. **F.** Western blot analysis on the expressions of CHOP and caspase3 cleavage from respective tumor tissue lysates. GAPDH was used as protein loading control. **G.** Tumor sections were stained with an anti-Ki-67 Ab to detect proliferating cells. A scale bar, 50 μm. **H.** TrxR1 enzyme activity of tumor tissue lysates was measured by endpoint insulin reduction assay. MDA levels of tumor tissue proteins extracted from xenografts.

We further observed the profile of proteins involved in mitochondrial apoptosis. Figure 6C and 6D showed that the activity of caspase 9 and caspase 3 was also dose-dependently increased following treatment with B19 in SGC-7901 cells, which were significantly blocked by pre-treatment with NAC (5 mM). Bcl-2 family members have been reported to be crucial regulators of the mitochondrial pathway. As shown in Figure 7A, western blots showed that B19 dose-dependently decreased the expression of anti-apoptotic proteins Bcl-2 and increased the expression of pro-apoptotic proteins Bax in three gastric cancer cell lines (SGC-7901, BGC-823 and KATO III). Pre-incubation with NAC almost totally attenuated these changes. Taken together, these data confirmed that B19 induces ROS-mediated mitochondrial dysfunction and apoptosis.

### B19 inhibited SGC-7901 xenograft tumor growth accompanied with increased ROS level and decreased TrxR1 activity in tumor tissues

On the basis of the in vitro data above, we investigated the effects of B19 on xenograft tumor growth in vivo. The nude mice with SGC-7901 xenografts were treated via daily ip injection of B19 at doses of 4 and 8 mg/kg or vehicle once the tumor had grown to a volume of 100-200 mm^3^. As shown in Figure 7A–7C, treatment with B19 at 4mg/kg and 8 mg/kg for 10 days dose-dependently resulted in significant reduction in both tumor volume and weight. In addition, there was no significant difference in body weight change among the vehicle group and B19-treated groups, suggesting that B19 exhibited no significant toxicity within the 10-day treatment (Figure 7D). The nontoxicity was further confirmed by H&E staining analysis in mouse kidneys, livers and hearts (Figure 7E). Western blotting analyses of the tumor tissues revealed that B19 treatment increased the levels of CHOP and cleaved caspase-3 in a dose-dependent manner (Figure 7F), indicating that B19-induced apoptosis in SGC-7901 cells is associated with activation of ER-stress in vivo. Ki-67 staining on tumor tissues showed that Ki-67 expression was inhibited by B19 administration (Figure 7G). We observed that TrxR1 activity in tumor tissue lysates significantly decreased by B19 administration (Figure 7H). MDA, a lipid peroxidation product, is a marker of cellular and tissular oxidative damage. We found that B19 elevated the level of lipid peroxidation product (MDA) in tumor tissues (Figure 7H). Combined with cytological data, these animal data revealed that B19 exhibits ROS-related anti-tumor activity and high safety in vivo.

## DISCUSSION

In our previous studies, curcumin derivative B19 exhibited high bioavailability and great anticancer effect in human non-small-cell lung cancer cells and human ovarian cancer cells [20, 31]. Also, we demonstrated that B19 induces cancer cell apoptosis via activation of endoplasmic reticulum stress signaling pathway [20, 31]. In this work, we demonstrated the upstream mechanism and the molecular target of B19 for its anti-cancer effects.

Firstly, we showed that B19 induced the accumulation of intracellular ROS in a dose- and time-dependent manner. Furthermore, B19 induced ROS-dependent apoptosis and cell cycle arrest in three human gastric cancer cell lines, accompanied with the corresponding changes in the levels of proteins involved in apoptosis- and cell cycle- related cascades. In addition to ER stress, ROS over production could lead to mitochondrial dysfunction, induce the depolarization of the mitochondrial membrane, provoke the release of cytochrome C from mitochondria into cytosol, which eventually results in an increase in the level of other pro-apoptotic molecules in the cytosol [33]. Mitochondrial membrane permeabilization is a central process in programmed cell death pathways and is regulated by Bcl- 2 family members via multiple molecular mechanisms. Using electron microscopy, we observed that the mitochondria in the B19-treated gastric cancer cells looked abnormally enlarged or swollen. In consistent with the morphological changes, B19 decreased Δψm, decreased the antiapoptotic/proapoptotic (Bcl-2/Bax) protein ratio and up-regulated the activity of caspase9 followed by caspase-3 activation in gastric cancer cells. More importantly, NAC fully reversed these changes above induced by B19 in human gastric cancer cells. Besides the cellular effects, xenografted mice alone showed increased tumor growth, while treatment with B19 could cause oxidative stress damage in the xenografted tumors. Together, these data substantiated the notion that ROS production plays a crucial role in B19's anti-cancer actions in human gastric cancer cells and also acts as upstream signaling molecules involved in B19-induced activation of mitochondrial pathway.

ER plays a critical role in the regulation of protein synthesis, folding and trafficking. Many signals have been reported to disrupt the ER function and induce ER stress. In addition, the role of ROS in ER-stress-mediated apoptosis has been demonstrated in a variety of cell types [34–36]. Our previous studies have reported that B19 was capable of inducing ER stress [20]. In this study, we revealed that B19 treatment concomitantly induces ROS-mediated ER stress response. Further, we observed extensive distension in the ER of SGC-7901 cells treated with B19. As expected, NAC pretreatment fully reversed these changes in ER induced by B19. These data indicate that ROS production is the critical upstream regulator of B19-induced ER stress in gastric cancer cells.

The thioredoxin system plays a crucial role in the redox regulation of multiple intracellular processes, involving in a number of physiological and pathological signaling pathways [37, 38]. This system regulates DNA replication and repair, protein synthesis and folding, and intracellular redox balance by counteracting ROS, and eventually influences cell growth, differentiation, and death [39]. As a component of the thioredoxin system, TrxR1 has been evidenced to be over-expressed and constitutively active in various kinds of cancer cells, enhance cancer cells proliferation, and exhibit pro-survival signaling which attributes to inciting a pro-survival effect in cancer cells [40]. Considering the important functions of TrxR1 in cancer development and progression, TrxR1 is now major target for anticancer drugs. Thus, the past years have witnessed increasing attention to developing novel inhibitors of this system as potential antitumor agents. In the present study, we identified TrxR1 as a target of B19 and revealed that B19 induces cancer cell by inhibiting this enzyme. Targeting TrxR1by B19 inhibits the physiological functions of TrxR1, but further turns the enzyme to an NADPH oxidase to directly generate superoxide anions, which leads to ROS accumulation within cells, consequently causing intracellular thiol depletion and finally eliciting oxidative stress. Both the direct inhibition of B19 on the cell-free TrxR1 activity and silencing TrxR1 by specific siRNAs further validated the result. We also searched the possible binding sites throughout the surface of TrxR1 using the molecular docking method, indicating Cys-498 is a key residue for the interaction of B19 and TrxR1.

**Figure 8:**
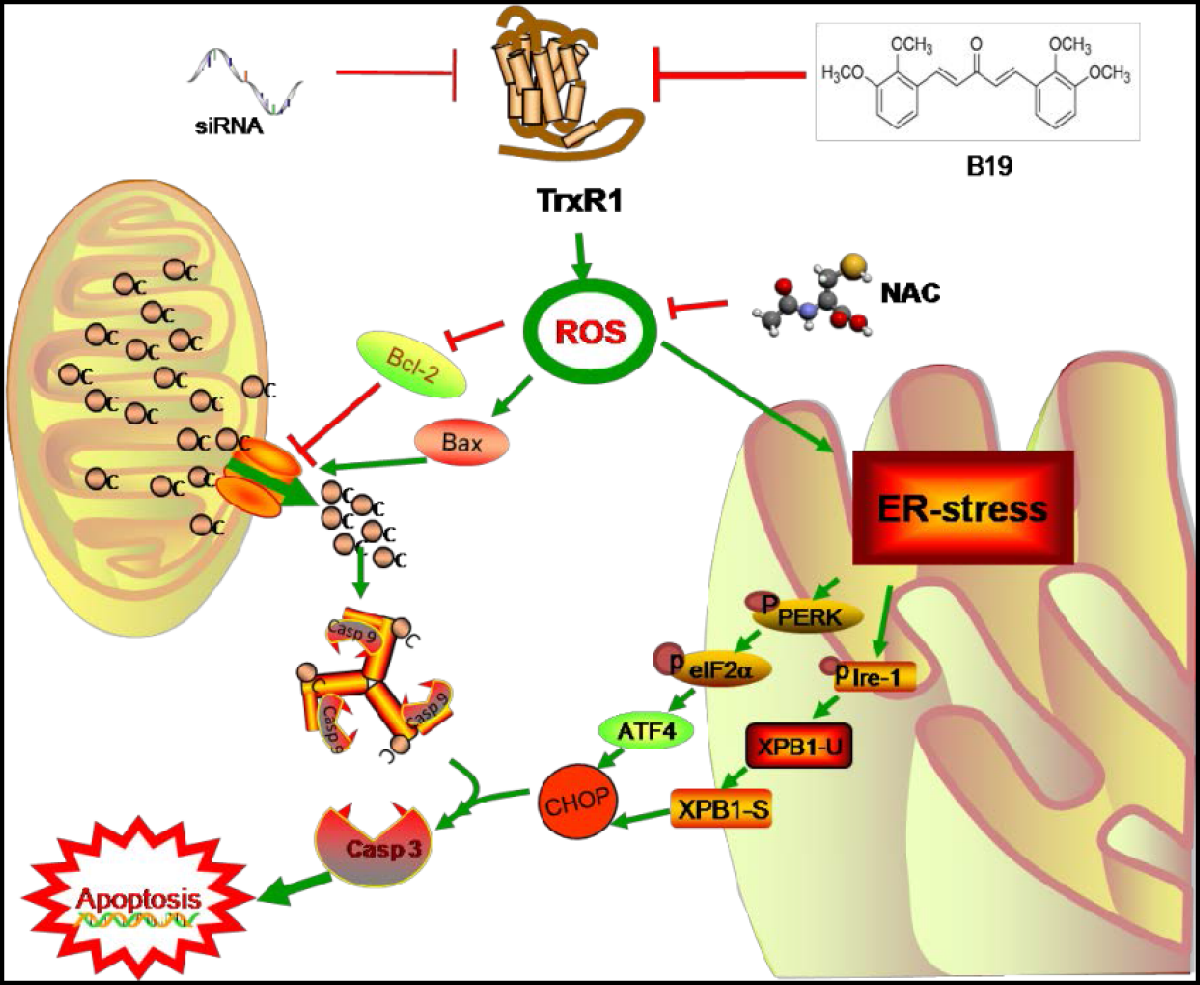
Schematic illustration of the underlying mechanism of B19's anti-cancer activity

Based on the analysis of experimental results, we summarized a scheme of the possible mechanisms involved in B19-induced cell death in human gastric cancer cells (Figure 8). In conclusion, we have discovered that TrxR1 is the target of B19in vitro, and demonstrated that B19 induces apoptotic cell death through ROS-mediated ER-stress and mitochondrial dysfunction pathway. The discovery of B19-TrxR1 interaction provides deep insight into the understanding of how B19 acts in vivo as well as developing the novel thioredoxin system-targeting small molecules for gastric cancer chemotherapy.

## MATERIALS AND METHODS

### Chemistry

B19, a monocarbonyl analogue of curcumin, was synthesized by direct aldol condensation of substituted benzaldehyde with acetone or cyclopentanone in alkaline media and was structurally identified by using MS and 1H NMR analysis, as described in our previous paper. Before use in biological experiments, this compound was recrystallized from CHCl3/EtOH and HPLC was used to determine their purity (99.56%). The structure is shown in Figure 1A.

### Cell culture and reagents

Human gastric cancer cell lines SGC-7901, BGC- 823 and KATO III were purchased from the Institute of Biochemistry and Cell Biology, Chinese Academy of Sciences. The cells were routinely cultured in RPMI 1640 medium (Gibco, Eggenstein, Germany) containing 10% heat-inactivated fetal bovine serum (Hyclone, Logan, UT), 100 units/ml penicillin, and 100 ug/ml streptomycin in a humidified cell incubator with an atmosphere of 5% CO2 at 37°C. FITC Annexin V apoptosis Detection Kit I and propidium iodide (PI) were purchased from BD Pharmingen (Franklin Lakes, NJ). 2′,7′-dichlorodihydrofluorescein diacetate (DCFH-DA) and JC-1 were purchased from Invitrogen (Carlsbad, CA). Antibodies including anti-Cdc2, anti-XBP-1, a'nti-Bcl-2, anti-Bax, anti-Cyclin B1, anti-cleaved PARP, anti-MDM-2, anti-p53, anti-TrxR1, anti-Ki67, anti-GAPDH, goat anti-mouse IgG-HRP, donkey anti-rabbit IgG-HRP and donkey anti-goat IgG-HRP were purchased from Santa Cruz Biotechnology (Santa Cruz, CA). Antibodies including anti-ATF-4, anti-p-EIF2α, anti-CHOP, anti-Cle-caspase3, were purchased from Cell Signaling Technology (Danvers, MA).

### MTT (MethylThiazolylTetrazolium) assay

Cells were seeded into 96-well plates at a density of 8×10^3^ per well and allowed to attach overnight in RPMI 1640 containing 10% heat-inactivated FBS. B19 was dissolved in DMSO and diluted with 1640 medium to final concentrations of 1.25, 2.5, 5, 7.5, 10, 12.5, 15 and 20 μM. The tumor cells were incubated with B19 for 24 h before the MTT assay.

### Determination of cellular reactive oxygen species(ROS)

Cellular ROS contents were measured by flow cytometry. Briefly, 5 × 10^5^ cells were plated on 60-mm dishes, allowed to attach overnight, and then treated with B19 (5, 10 or 20μM) in the presence or absence of NAC (5 mM) for 1 h. After removal of the medium, the ROS indicator DCFH-DA (10 μM) in fresh FBS-free medium was added and incubation continued for 30 min at 37°C in the dark. Cells were collected and the fluorescence was analyzed using a FACSCalibur flow cytometer (BD Biosciences, CA). Meanwhile, the levels of intracellular ROS of cells were also determined by a fluorescence microscope (Nikon, Japan).

### Cell apoptosis analysis

SGC-7901, BGC-823 and KATO III cells were plated on 6-well plates for 12 h, and then treated with B19 (5.0, 10 or 20 μM) for 24 h in the presence or absence of NAC (5 mM). Cells were then harvested, washed twice with ice-cold PBS, and evaluated for apoptosis by double staining with FITC conjugated Annexin V and Propidium Iodide (PI) in binding buffer for 30 min using a FACSCalibur flow cytometer (BD Biosciences, CA).

### Cell cycle analysis

SGC-7901, BGC-823 and KATO III cells were placed on 6-well plates for 24 h, and then treated with B19 (5, 10 or 20 μM) for 15 h in the presence or absence of NAC (5 mM). Cells were then collected into flow cytometry tubes and centrifuged at 1,000 rpm for 5 min to obtain cell pellets. The supernatant was discarded, and the cells were washed with ice-cold PBS and then re-centrifuged. The cells were resuspended in 100 μL PBS, 3 mL of −20°C ice-cold 75% ethanol was added, and the cells were then incubated for 4 h at −20°C. The cells were washed twice with PBS. The DNA was labeled with propidium iodide at a final concentration of 0.05 mg/mL and incubated at 4°C for 20 min in the dark. Cell cycle analysis was performed with FACSCalibur flow cytometer (BD Biosciences, CA).

### Western blotting analysis

Cells or tumor tissues were homogenized in protein lysate buffer, and debris was removed by centrifugation at 12,000 rpm for 10 min at 4°C. The protein concentrations in all samples were determined by using the Bradford protein assay kit (Bio-Rad, Hercules, CA). After addition of sample loading buffer, protein samples were electrophoresed and then transferred to poly-vinylidenedifluoride transfer membranes. The blots were blocked for 2 h at room temperature with fresh 5% nonfat milk in TBST and then incubated with specific primary antibody in TBST overnight at 4°C. Following three washes with TBST, the blots were incubated with horseradish peroxidase-conjugated secondary antibodies for 1 h, and the immunoreactive bands were visualized by using ECL kit (Bio-Rad, Hercules, CA). The density of the immunoreactive bands was analyzed using Image J computer software (National Institute of Health, MD).

### Endpoint insulin reduction assay

Untreated gastric cancer SGC-7901 cells or tumor tissues from genografts model were collected and lysed with RIPA buffer in the presence of protease inhibitors. The concentration of the protein in the cell lysate and tumor tissue lysate was quantified using the Bradford method. A volume corresponding to 50 μg of total proteins from cell extract was incubated with various concentrations of B19 (5, 10, 20,40 and 50 μM) for 2 h at room temperature. A master mixture of TE buffer (50 μl) containing 4 μM E. coli Trx, 0.4 mM NADPH, and 0.32 mM insulin was added to the cell lysate or tumor tissue lysate (a volume corresponding to 50 μg of total proteins). Then the reaction mixtures were incubated at room temperature for 0.5 h. The reaction was terminated by addition of 100 μl of 1 mM DTNB in 6 Mguanidinehydrochloride (pH 8.0) and the absorbance was measured using a microplate reader at 412 nm. The same amounts of DMSO (0.05%, v/v) were added to the control experiments and the activity was expressed as a percentage of the control.

### Molecular docking simulation

To further study the interaction between the B19 and TrxR1, a covalent dock was implemented by CovalentDock. The crystal structure of human TrxR1 (PDB code 2ZZ0, chain A) was used for present docking study. The center coordination of dock pocket was set as −29.11, −1.26, and −6.55 which calculated by selecting residue Cys-497, Cys-498 and W407. A grid box size of 60×60×60 points with a spacing of 0.375 Å between the grid points was implemented. The default parameters were used for running the docking simulation.

### Transient transfection of small interfering RNA (siRNA)

The siRNA duplexes used in this study were purchased from Invitrogen (Carlsbad, CA, USA) and Sigma-Aldrich (St. Louis, MO, USA). They have the following sequences: The sequence1 of TrxR1 (The forward sequence: 5′-GCAAGACUCUCGAAAUUAUdTdT-3′, the reverse sequence: 5′-AUAAUUUCGAGAGUCUUGCdAdG-3′), The sequence2 (The forward sequence: 5′-GUUCGUACC AAUUAAAGUUdTdT-3′, the reverse sequence: 5′-AACU UUAAUUGGUACGAACdTdT-3′). Negative Universal Control (Invitrogen) was used as the control. SGC-7901 cells (3×10^5^/well) were seeded into 6 well plates and cultured for 24 h, and then were transfected with siRNA duplexes against human TrxR1 (100 nM) or control siRNA by lipofectamine 2000 (Invitrogen) according to manufacturer's protocol. Cells were further incubated for 48 h before harvest for detection of TrxR1 expression by Western blot.

### Electron microscopy

SGC-7901 cells were treated with vehicle control (DMSO) or B19 at the dose of 20 μM in the presence or absence of NAC (5 mM)for indicated time in 60 mm plates. Then the cells were collected and fixed in phosphate buffer (pH 7.4) containing 2.5% glutaraldehyde overnight at 4°C. The cells were postfixed in 1% OsO4 at room temperature for 60 min, stained with 1%uranyl acetate, dehydrated through graded acetone solutions, and embedded in Epon. Aeras containing cells were block mounted and cut into 70 nm sections and examined with the electron microscope (H-7500, Hitachi, Ibaraki, Japan).

### Evaluation of mitochondrial membrane potential (Δψm)

The effects of B19 on the cell mitochondrial membrane potential (Δ*ψ*_m_) were examined by fluorescence microscope using JC-1as specific probe. Cells were treated with B19 for 12 h and stained with JC-1 in a humidified atmosphere of 5% CO_2_ at 37°C for 30 minutes. Images acquired by using the Nikon fluorescence microscope (40X amplification, Nikon, Japan).

### Determination of caspase-3/9 activity

Caspase-3/9 activity in cell lysates was determined using a Caspase-3/9 activity kit (Beyotime Institute of Biotechnology, Nantong, China) according to the manufacturer's protocol. The caspase-3/9 activity was normalized by the protein concentration of the corresponding cell lysate and expressed as percentage of treated cells to that of control.

### Tumor growth in nude mice

All animal experiments were complied with the Wenzhou Medical University's Policy on the Care and Use of Laboratory Animals. Protocols for animal studies were approved by the Wenzhou Medical College Animal Policy and Welfare Committee (Approved documents: 2012/APWC/0216). Five-week-old athymic BALB/cA nu/nu female mice (18-22 g) purchased from Vital River Laboratories (Beijing, China) were used for in vivo experiments. Animals were housed at a constant room temperature with a 12/12-hr light/dark cycle and fed a standard rodent diet and water. The mice were divided into three experimental groups with six mice in each group. SGC-7901 cells (1×10^6^) in 0.1 ml PBS was injected subcutaneously into the right flank of each mouse. When tumors reach a volume of 100-200 mm^3^ on all mice on day 10, treated mice were intraperitoneally (ip) injected with a water-soluble preparation of compound 19 in PBS at a dosage of 4 or 8 mg/kg, whereas control mice were injected with liposome vehicle in PBS. The tumor volumes were determined by measuring length (l) and width (w) and calculating volume (V = 0.5 × l × w^2^) at the indicated time points. At the end of treatment, the animals were sacrificed, and the tumors were removed and weighed for use in histology, and proteins expression studies.

### MDA assay

MDA content was assayed using Lipid Peroxidation MDA Assay kit following the manufacturer's instructions (Beyotime). MDA levels were detected using multimode microplate readers (MD, Sunnyvale, CA) at 532 nm.

### Immunohistochemistry and HE staining

The harvested tumor tissues were fixed in 10% formalin at room temperature, processed and embedded in paraffin. Parraffin-embedded tissues were sectioned (5 μm thick). Tissue sections were primarily stained with indicated antibodies. The signal was detected by biotinylated secondary antibodies, and developed in DAB. Quantity assay of the immunochemistry data was obtained with Image-Pro Plus 6.0 (Media Cybernetics, Inc, Bethesda, MD). For histologic analysis, the harvested heart, kidney and liver tissues were fixed in 4 % formaldehyde, dehydrated with an ethanol gradient, embedded in paraffin, and the paraffin tumor tissue sections (5μm) were stained with hematoxylin and eosin (H&E). Each image of the sections was captured using a light microscope (400× amplification, Nikon, Japan).

### Statistical analysis

All experiments were assayed in triplicate (n=3). Data are expressed as means ± SEM. All statistical analyses were performed using GraphPad Pro. Prism 5.0 (GraphPad, SanDiego, CA). Statistical differences between two groups were assessed by Student's t test. A p value <0.05 was considered statistically significant.

## Abbreviations

ROS: Reactive oxygen species
TrxR1: Thioredoxin reductase 1
NAC: N-acetyl-L-cysteine
ER: endoplasmic reticulum
CHOP: C/EBP-homologous protein
ATF-4: activating transcription factor 4
XBP-1: X-box binding proteins 1
p-EIF2α: phosphorylated α subunit of eukaryotic initiation factor 2
